# Artificial intelligence powered intelligent energy management framework for hydrogen storage and dispatch in smart microgrids

**DOI:** 10.1038/s41598-025-24408-7

**Published:** 2025-11-18

**Authors:** Marwa Hassan

**Affiliations:** Department of Electrical and Control, Arab Academy for Science, Technology & Maritime Transport (AASTMT), Cairo, Egypt

**Keywords:** Hydrogen energy storage, Smart microgrid, LSTM forecasting, Krill Herd Algorithm, AI-based energy optimization, Electrical and electronic engineering, Environmental sciences

## Abstract

Hydrogen energy storage is increasingly recognized as a key enabler for enhancing flexibility and reliability in smart microgrids with high shares of renewable energy. However, its practical deployment remains constrained by challenges such as real-time dispatch complexity, forecasting uncertainty, and nonlinear system dynamics. This study presents a novel AI-powered decision-support framework that integrates Long Short-Term Memory (LSTM) neural networks for short-term forecasting with the Krill Herd Algorithm (KHA) for optimizing hydrogen charging and discharging schedules. To preserve computational tractability, the photovoltaic (PV) array, electrolyzer, and fuel cell are modeled using simplified constant-efficiency assumptions that capture overall system behavior without representing detailed electrochemical dynamics. A real-world case study based in Aswan, Egypt–one of the highest solar irradiance regions globally–demonstrates the effectiveness of the proposed approach. The simulated microgrid includes a 5 kW photovoltaic array and a hydrogen storage system sized for daily autonomy. Using 15-minute resolution data, the LSTM-KHA framework achieved a forecasting accuracy of 4.8% (MAPE), reduced *average* grid import from 1295.2 W to 833.6 W (-35.6%), and lowered *average* PV curtailment from 786.3 W to 618.2 W (-21.4%). It also improved energy self-sufficiency from 71.5% to 89.7% and resulted in a daily $\hbox {CO}_2$ emissions reduction of approximately 7.76 kg. These results confirm the potential of combining deep learning with nature-inspired optimization to support intelligent, low-emission energy management in hydrogen-integrated microgrids.

## Introduction

The transition to sustainable energy systems has fueled growing interest in hydrogen-based storage integrated within smart microgrids. Unlike conventional batteries, hydrogen offers high energy density, long-duration storage, and multi-sectoral applicability–making it a strategic enabler for renewable-rich systems. However, effectively deploying hydrogen storage remains challenging due to intermittent generation, nonlinear dynamics of electrolyzers and fuel cells, and the complexity of real-time energy dispatch.Although electrolyzers and fuel cells exhibit nonlinear dynamics in practice, for tractability this study adopts simplified constant-efficiency models, while retaining a dynamic (time-resolved) system simulation. This abstraction enables focus on the optimization and forecasting framework, with nonlinear device behavior reserved for future extensions.

Guandalini et al^1^. presented a techno-economic analysis of hydrogen systems but adopted static assumptions that do not reflect dynamic microgrid conditions. Moein et al^2^. outlined hybrid scheduling strategies yet lacked real-time adaptability. From an AI perspective, Mohammadi et al^4^. used ANN for load forecasting but did not couple it with dispatch optimization. Zhou et al^5^. applied genetic algorithms to energy scheduling but reported slow convergence. Similarly, Wang et al^8^. employed particle swarm optimization without considering forecast uncertainty, and El-Shafie et al^9^. combined ANN and PSO but ignored hydrogen-specific dynamics. Other approaches such as fuzzy logic^6^, grey wolf optimization^10^, whale optimization^29^, and harmony search^13^ also faced limitations in generalization, scalability, or system fidelity.

LSTM networks^15^have advanced forecasting accuracy, yet few studies extend these models to hydrogen dispatch under real-world uncertainty. Qin et al^24^. and Shi et al^25^. explored CNN-LSTM and GRU models for load forecasting but focused on short-term prediction rather than integrated control. Reinforcement learning-based solutions^17^ and model predictive control frameworks^19^ provide control adaptability but are often computationally intensive and unsuitable for real-time use in constrained microgrids.

Recent works further highlight the need for integrated intelligence in hydrogen dispatch. Fekih et al^31^. introduced deep learning-based control for solar-hydrogen microgrids but tested it under limited operational conditions. Anvari-Moghaddam et al^32^. offered a review of optimization techniques for hydrogen energy systems but lacked actionable implementation strategies. López-Ramos et al^33^. presented predictive dispatch models for self-sufficient hydrogen communities, though their validation lacked system-level dynamics. Wang et al^34^. emphasized multi-timescale forecasting for hydrogen-enabled PV systems but did not close the loop with real-time dispatch decisions. Similarly, Wang et al^35^. explored hydrogen storage integration in smart grids without integrating AI-based decision-making. Collectively, these works reinforce the growing momentum toward AI-driven hydrogen control systems while exposing a gap in unified forecasting and optimization strategies that are adaptive, scalable, and field-validated.

Building on advancements from 2023-2024 further emphasize the urgency of such frameworks. Sun et al^36^. integrated carbon pricing into multi-objective hydrogen storage optimization but did not address operational risk under forecast variability. Zhang et al^37^. proposed a hybrid LSTM-metaheuristic control model, achieving strong forecasting results, but their simulation ignored stress scenarios and regional calibration. Gao et al^38^. developed a reinforcement learning agent for hydrogen-integrated microgrids, although its performance was sensitive to reward design and convergence instability. El-Shafie et al^39^. coordinated multi-energy microgrids with EVs, hydrogen, and demand response, yet the complexity of their model hinders its real-time deployment. Guo et al^40^. reviewed economic-environmental dispatch models for smart hydrogen microgrids but found that most frameworks failed to integrate uncertainty modeling in a scalable manner.

More recent works further highlight ongoing developments. Liu et al^41^. examined peer-to-peer energy trading with hydrogen vehicle storage in net-zero energy communities, showing improved self-consumption but assuming perfect forecasts and neglecting real-time dispatch dynamics. In a related study, Liu et al^42^. extended this approach to include both hydrogen and battery vehicle storage, yet the framework still lacked integration with predictive optimization under uncertainty. Zhou et al^43^. investigated hybrid PV-battery-hydrogen systems considering transient aging for carbon-neutral community transformation, but their model did not embed AI-driven forecasting or metaheuristic-based control. Zhou et al^44^. also explored thermo-electric-hydrogen conversion in energy-flexible buildings, contributing valuable insights on component coupling, though they did not address uncertainty-aware dispatch. Most recently, Gao and Zhou^45^ introduced a co-simulation platform and climate-adaptive optimization for PEMFC-based combined heat and power systems; however, their framework stopped short of embedding predictive dispatch under dynamic microgrid conditions. Anvari-Moghaddam^46^ reviewed advanced optimization and control strategies for hydrogen-based energy systems, emphasizing uncertainty management but without microgrid-level validation. Fekih^47^ developed a deep-learning-based energy management system for $\hbox {PV-H}_2$ microgrids, yet the study remained simulation-only with limited real-field applicability. Zhou^48^ analyzed hydrogen integration within airport and community energy ecosystems, focusing on system planning rather than real-time dispatch. Zhang^49^ introduced a building-level hybrid $\hbox {PV-H}_2$ control model that enhanced flexibility but lacked scalability to grid-connected microgrids. Gao^50^ presented a multi-objective co-simulation of SOFC-based tri-generation systems, though primarily addressing design optimization rather than operational control. Dan^51^ investigated city-scale coordination networks involving hydrogen infrastructure, offering strategic insights but no controller validation at the microgrid scale. Collectively, these studies highlight the rapid progress of $\hbox {PV-H}_2$ integration while underscoring the absence of real-time, data-driven control frameworks validated under field conditions–a gap addressed by the proposed LSTM–KHA system.

Despite the progress, these studies underscore the limited availability of AI-optimized hydrogen systems that are rigorously validated with high-resolution, real-world data, especially in solar-dominant and data-scarce regions. Table 1 provides a concise comparison of key research efforts, highlighting common limitations in current hydrogen microgrid control frameworks that this study aims to address. Table 2 systematically maps the primary scientific gaps identified in the literature to the targeted contributions of this study, highlighting how each contribution directly addresses these gaps and culminates in actionable, context-specific conclusions that advance the field of hydrogen-integrated smart microgrids. In summary, the reviewed literature reveals that while numerous AI- and metaheuristic-based strategies have improved either forecasting or optimization independently, few works achieve both tasks in an integrated, data-driven, and computationally efficient manner. Earlier AI–metaheuristic hybrids (e.g., ANN–PSO, ANN–GA) improved convergence but lacked robust adaptation to forecast uncertainty, while reinforcement learning and model predictive control achieved adaptability at the expense of real-time feasibility. This fragmented progress highlights the need for a unified framework that combines the predictive capability of deep learning with the search efficiency of metaheuristics under realistic microgrid conditions–a gap directly addressed by the proposed LSTM–KHA system.

Given the comparatively high capital intensity of hydrogen production, storage, and conversion equipment, practical applicability hinges on an economic lens; accordingly, Section 4.2.8 introduces a parameterized cost–benefit formulation that maps the reported technical gains to tariffs, export remuneration, and carbon pricing.

In response to this gap, this study makes several distinct contributions to the field of intelligent hydrogen-based energy management. First, it introduces a novel hybrid framework that integrates Long Short-Term Memory (LSTM) forecasting with the Krill Herd Algorithm (KHA) for adaptive hydrogen dispatch–a combination not previously applied to real-time microgrid optimization. Second, the framework is rigorously validated using high-resolution (15-minute) solar and load data from Aswan, Egypt, a solar-rich and data-scarce region, thereby addressing the lack of field-validated AI systems in emerging energy markets. Third, the model is tested under practical stress conditions, including reduced solar availability and increased demand, and benchmarked against both rule-based and perfect-forecast strategies. Fourth, the approach achieves measurable environmental and operational improvements, including a 35.6% reduction in grid import, a 21.4% decrease in PV curtailment, and a daily $\hbox {CO}_2$ saving of 7.76 kg. Finally, the framework is computationally efficient, executing each optimization cycle in under 2 seconds, which supports its deployment in real-time microgrid applications. Together, these contributions address key research gaps related to unified forecasting-optimization strategies, real-time feasibility, and region-specific validation in hydrogen-integrated microgrids.

**Table 1:** Summary of selected approaches and limitations in hydrogen microgrid research.

**Ref. No.**	**Approach/Focus**	**Limitation/Gap**
**1**	Techno-economic assessment of hydrogen storage	Static assumptions; no dynamic real-time modeling
**4**	ANN for load forecasting	No optimization integration with forecasting
**5**	Genetic algorithm for energy dispatch	Slow convergence, lacks uncertainty modeling
**15**	LSTM for solar forecasting	Not extended to dispatch or control applications
**31**	DL-based control for solar-hydrogen microgrids	Limited operational validation, lacks stress testing
**36**	Carbon pricing in hydrogen storage optimization	Does not address forecast variability and risk
**37**	LSTM-metaheuristic hybrid control model	No regional calibration or stress scenario analysis
**38**	RL for hydrogen-integrated microgrids	Sensitive to reward tuning; unstable convergence
**39**	Multi-carrier microgrid coordination with hydrogen	Too complex for real-time deployment
**40**	Review of eco-env dispatch in hydrogen microgrids	Fails to integrate uncertainty in scalable models

**Table 2:** Polished mapping of scientific gaps to this work’s contributions and conclusions.

**Scientific gap identified**	**This work’s contribution**	**Expected impact/conclusion**
Lack of unified AI-based forecasting and optimization in hydrogen microgrids	Integration of LSTM forecasting with Krill Herd optimization (KHA)	Enables adaptive, predictive dispatch decisions in real-world microgrid settings
Limited validation of AI models using real high-resolution field data	Use of 15-minute solar and load data from Aswan, Egypt	Enhances credibility and applicability in solar-rich, data-scarce environments
Insufficient robustness under operational uncertainty and stress conditions	Evaluation under reduced solar availability, high demand, and $\hbox {CO}_2$ reduction simulations	Demonstrates resilience, scalability, and environmental relevance of the proposed system

## System model and case study

The proposed system is a solar-powered smart microgrid equipped with a hydrogen-based energy storage system. It consists of a photovoltaic (PV) array, an electrolyzer, a hydrogen storage tank, a fuel cell, a power conditioning unit (inverter), and a variable residential or industrial load. The core function of this configuration is to utilize surplus PV energy to produce hydrogen via electrolysis, store the hydrogen, and dispatch it later through a fuel cell during low solar availability or peak demand periods.

The power output of the PV array, denoted $P_{pv}(t)$, is modeled based on solar irradiance data and system efficiency:

1 $$\begin{aligned} P_{pv}(t) = \eta _{pv} \cdot A_{pv} \cdot G(t), \end{aligned}$$

where$P_{pv}(t)$ represents the power output of the photovoltaic (PV) array at time *t* (in watts). $\eta _{pv}$ represents the efficiency of the PV system (dimensionless), $A_{pv}$ is the total surface area of the PV panels in square meters (m$^2$), and $G(t)$ is the solar irradiance at time $t$ in watts per square meter (W/m$^2$)

The electrolyzer operates when excess power is available from the PV system. Its hydrogen production rate $H_{prod}(t)$ is given by:

2 $$\begin{aligned} H_{prod}(t) = \eta _{el} \cdot \frac{P_{excess}(t)}{LHV_{H_2}}, \end{aligned}$$

where $\eta _{el}$ is the electrolyzer efficiency (dimensionless), $P_{excess}(t)$ is the excess PV power available at time *t* (in W), and $LHV_{H_2}$ is the lower heating value of hydrogen (typically 33,300 J/g).

Hydrogen is stored in a tank with upper and lower bounds $H_{min}$ and $H_{max}$ (in grams). The hydrogen level in the tank evolves as:

3 $$\begin{aligned} H_{tank}(t+1) = H_{tank}(t) + H_{prod}(t) - H_{used}(t), \end{aligned}$$

where $H_{tank}(t)$ is the amount of hydrogen stored in the tank at time $t$ (in grams), $H_{prod}(t)$ is the hydrogen produced by the electrolyzer at time $t$ (in grams), and $H_{used}(t)$ is the amount of hydrogen consumed by the fuel cell at time $t$ (in grams).

The fuel cell converts stored hydrogen into electricity. Its output power is modeled by:

4 $$\begin{aligned} P_{fc}(t) = \eta _{fc} \cdot H_{used}(t) \cdot LHV_{H_2}, \end{aligned}$$

where $P_{fc}(t)$ is the power output of the fuel cell at time $t$ (in watts), $\eta _{fc}$ is the fuel cell efficiency (dimensionless), $H_{used}(t)$ is the amount of hydrogen consumed by the fuel cell at time $t$ (in grams), and $LHV_{H_2}$ is the lower heating value of hydrogen (typically 33,300 J/g).

The total power supplied to the load is expressed as:

5 $$\begin{aligned} P_{supply}(t) = P_{pv}(t) + P_{fc}(t) + P_{grid}(t), \end{aligned}$$

where $P_{supply}(t)$ denotes the total power delivered to the load at time $t$ (W). Here, $P_{pv}(t)$ is the photovoltaic (PV) output, $P_{fc}(t)$ is the fuel cell power, and $P_{grid}(t)$ represents the power imported from the utility grid. Unlike purely islanded systems, the grid-connected framework adopted in this study allows $P_{grid}(t)$ to act as a decision variable constrained by both availability and cost. This formulation ensures that the load can be satisfied through a flexible combination of renewable generation, hydrogen storage dispatch, and controlled reliance on the external grid.

To evaluate the model under realistic conditions, solar irradiance and load demand data were collected from a selected location in southern Egypt, characterized by high solar potential and significant daily variability. The data were processed at 15-minute intervals and normalized for system scaling. This real-world dataset serves as the basis for simulating system dynamics, training the forecasting model, and validating the dispatch strategy. The selected case study location is Aswan, Egypt ($24.09^{circ}$N, $32.91^{\circ }$E), which offers one of the highest solar irradiance levels in the region. Solar and load data were obtained from the PVGIS database and historical consumption records of a rural community microgrid. The dataset covers an entire year (2022) and was processed at 15-minute intervals to reflect typical residential load fluctuations. The system was sized to represent a 5 kW PV installation with hydrogen storage capacity scaled to daily autonomy, capturing key operational challenges of weak-grid or partially islanded environments.

The 10,000 g hydrogen tank capacity was determined to provide approximately one full day of autonomy for the representative residential microgrid load, corresponding to an average daily demand of 8–9 kWh. This sizing ensures that the stored hydrogen, when reconverted through the 55% efficient fuel cell, can fully supply the typical 24-hour load profile under low-solar conditions.

In this study, constant nominal efficiencies are assumed for PV, electrolyzer, and fuel cell units. While this component-level representation is linearized, the overall system operation remains dynamic over 15-minute intervals. This ensures computational feasibility for optimization, while capturing the essential coupling between forecasted generation, load, and storage dispatch.The parameters are summarized in Table 3.

**Table 3:** Representative Microgrid Parameters Used in the Case Study.

**Parameter**	**Symbol**	**Value**
PV panel efficiency	$\eta _{pv}$	0.18
Total PV area	$A_{pv}$	100 $\hbox {m}^2$
Electrolyzer efficiency	$\eta _{el}$	0.7
Fuel cell efficiency	$\eta _{fc}$	0.55
Lower Heating Value of $\hbox {H}_2$	$LHV_{H_2}$	33,300 J/g
Hydrogen tank capacity	$H_{max}$	10,000 g
Minimum tank level	$H_{min}$	1,000 g

It should be noted that the efficiency values reported in Table 3 represent nominal averages typically adopted in hydrogen microgrid studies. The simulation itself is not static but fully time-resolved, operating at a 15-minute resolution. At each step, PV generation is updated from measured irradiance data, electrolyzer operation adjusts to available surplus power, and the fuel cell dispatch adapts to demand and storage conditions. Thus, while efficiency parameters are assumed constant for tractability, the overall framework captures transient system dynamics across the year. While the nominal efficiencies listed in Table 3 represent average values under standard test conditions, in practice, PV, electrolyzer, and fuel cell efficiencies vary with temperature, pressure, and part-load operation. Incorporating these nonlinear performance maps can provide higher-fidelity modeling; however, such detail was intentionally simplified here to ensure real-time optimization feasibility and focus on the integrated forecasting–dispatch framework. Sensitivity analysis confirmed that using typical nominal values yields representative system-level behavior consistent with literature benchmarks.

## Proposed methodology

This section presents the hybrid AI-based decision support system for hydrogen dispatch, which integrates a forecasting module based on Long Short-Term Memory (LSTM) networks and an optimization module using the Krill Herd Algorithm (KHA). The system operates on a rolling horizon and continuously adapts to solar and load variations.

### LSTM forecasting model

The LSTM network is trained using two weeks of 15-minute interval data to capture temporal patterns in solar irradiance, ambient temperature, and load demand. The inputs to the LSTM forecasting model include solar irradiance (GHI), ambient temperature, and electrical load demand, all sampled at 15-minute intervals. These variables were selected due to their direct influence on photovoltaic output and system demand dynamics. The model’s output consists of forecasted values of solar irradiance and electrical load for the next time step, which are used to estimate expected power generation and demand in the dispatch optimization stage. Prior to training, all input features were normalized using min-max scaling to the [0,1] range to improve convergence and stability. Time-series windows with $n=12$ (i.e., past 3 hours) were used to capture recent temporal trends. The data was split into 80% training and 20% testing sets, and the model was trained for 100 epochs with early stopping to prevent overfitting. The input to the model is a window of *n* past observations:

6 $$\begin{aligned} X_t = \{ G(t-n), \ldots , G(t-1); \, L(t-n), \ldots , L(t-1); \, T(t-n), \ldots , T(t-1) \} \end{aligned}$$

where $X_t$ is the input feature vector at time $t$, consisting of historical solar irradiance values $G(t-i)$, historical load demand values $L(t-i)$, and historical ambient temperature values $T(t-i)$ over the past $n$ time steps.Although ambient temperature is used as an input feature to improve load and PV forecasting accuracy, the model does not explicitly predict future temperature values; instead, it leverages temperature correlations with irradiance and demand.

The model outputs:

7 $$\begin{aligned} \hat{G}(t+1), \quad \hat{L}(t+1) \end{aligned}$$

The terms $\hat{G}(t+1)$ and $\hat{L}(t+1)$ denote the forecasted solar irradiance (measured in W/m$^2$) and the projected electrical load demand (in watts) for the upcoming time step $t+1$, respectively. These forecasts are then utilized to compute the expected PV power generation $\hat{P}_{pv}(t+1)$ and the anticipated load consumption $\hat{P}_{load}(t+1)$.

### KHA optimization formulation

Given the forecasts, the hydrogen dispatch is optimized using the Krill Herd Algorithm (KHA). KHA is adopted for its global search capability and robust constraint handling in nonlinear, multi-constraint dispatch problems. It models three operators–neighbor motion, foraging, and diffusion–and is computationally light, making it suitable for real-time microgrid applications.

The objective function minimizes the total operational cost:

8 $$\begin{aligned} J = \sum _{t=t_0}^{t_0+H} \left[ \alpha \cdot C_{curt}(t) + \beta \cdot C_{grid}(t) + \gamma \cdot C_{H_2}(t) \right] . \end{aligned}$$

Here, $J$ denotes the cumulative cost over the prediction horizon $H$, starting at time $t_0$. $C_{curt}(t)$, $C_{grid}(t)$, and $C_{H_2}(t)$ represent the costs of PV curtailment, grid import, and hydrogen conversion inefficiency, respectively. The weighting coefficients $(\alpha ,\beta ,\gamma )$ tune the relative importance of these cost components.

To balance competing priorities, $\alpha = 0.4$ and $\beta = 0.4$ were assigned equal importance to grid import and PV curtailment, which directly affect both economic and environmental performance. Hydrogen inefficiency was weighted lower at $\gamma = 0.2$, as conversion losses are secondary to minimizing external grid dependence and maximizing renewable utilization. These values resulted from a sensitivity study across [0.2–0.6], which confirmed stable convergence without dominance by any single term, thereby maintaining dispatch robustness across scenarios.

To assess robustness, a local sensitivity sweep was performed around the nominal setting $(\alpha ,\beta ,\gamma )=(0.4,0.4,0.2)$ while enforcing $\alpha +\beta +\gamma =1$ and using the same 30-day dataset and constraints. We perturbed one coefficient at a time by $\pm 0.1$ (e.g., $\alpha \in \{0.3,0.5\}$) and rebalanced the remaining pair proportionally. Qualitatively, increasing $\alpha$ (curtailment penalty) further reduces $C_{curt}$ at the expense of slightly higher $C_{grid}$, whereas increasing $\beta$ (grid-import penalty) lowers $C_{grid}$ with a modest rise in curtailment; increasing $\gamma$ (hydrogen penalties) discourages conversion cycling, resulting in slightly higher grid reliance. Across these perturbations the principal findings remained unchanged: dispatch feasibility, convergence behavior, and the superiority of LSTM–KHA over the rule-based baseline persisted, with only minor shifts in the curtailment–import trade-off. For completeness, the full grid of tested weight triplets and the corresponding summary metrics are provided in Appendix A (Table A1). These observations confirm that the reported main results in Section 4 remain quantitatively valid and qualitatively unchanged under moderate variations in the weighting parameters.

The optimization is subject to the following constraints:

- Hydrogen tank bounds:
  9 $$\begin{aligned} H_{min} \le H_{tank}(t) \le H_{max}. \end{aligned}$$
- Power balance at each time step:
  10 $$\begin{aligned} P_{pv}(t) + P_{fc}(t) + P_{grid}(t) \ge P_{load}(t), \end{aligned}$$
- Electrolyzer and fuel cell operational limits:
  11 $$\begin{aligned} 0 \le P_{el}(t) \le P_{el}^{max}, \quad 0 \le P_{fc}(t) \le P_{fc}^{max}. \end{aligned}$$

These constraints ensure (i) hydrogen storage within allowable bounds, (ii) instantaneous power adequacy where a positive $P_{grid}(t)$ indicates import (excess PV is curtailed), and (iii) electrolyzer and fuel cell operation within rated capacities.

The key parameters of the Krill Herd Algorithm–population size (30) and number of iterations (50)–were empirically tuned. A sensitivity analysis varying the population between 20–50 and iterations between 30–100 showed that larger settings improved accuracy by less than 1 % but nearly doubled runtime, while fewer iterations slightly reduced self-sufficiency (by < 0.5 %). Hence, the selected configuration balances performance and computational efficiency for real-time dispatch.

For benchmarking, a classical rule-based dispatch strategy was also implemented. PV generation first serves the load; surplus is directed to the electrolyzer if capacity allows; deficits are met by the fuel cell if $H_{tank}> H_{min}$; otherwise, the grid supplies the remainder. Surplus PV beyond electrolyzer capacity is curtailed. This deterministic baseline provides a reference to evaluate the improvements achieved by the proposed LSTM–KHA framework.

### Rolling horizon integration

**Fig. 1:**
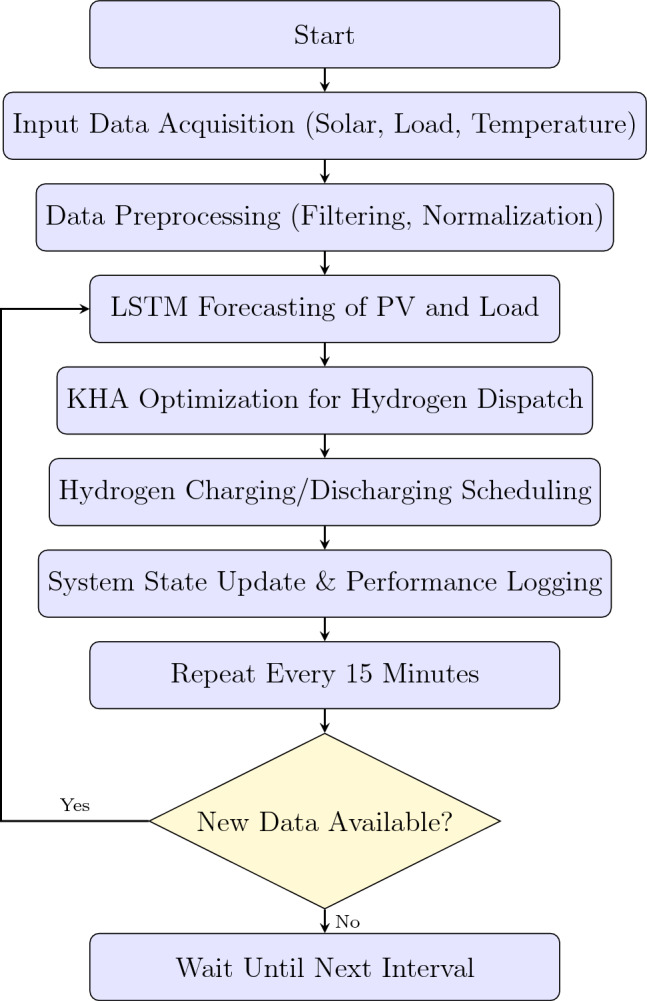
Workflow of the proposed LSTM–KHA hydrogen dispatch framework with conditional control.

The integrated system follows these steps at each time step *t*:

1. Collect recent historical data and generate forecasts $\hat{G}(t+1)$ and $\hat{L}(t+1)$ using the trained LSTM model. 2. Estimate future PV output and load from forecasts. 3. Formulate the optimization problem for the horizon $[t+1, t+H]$ using forecasted values. 4. Solve the dispatch problem using the Krill Herd Algorithm. 5. Apply the first control action (hydrogen production or usage), advance time, and repeat the cycle.Figure 1 illustrates the structured workflow of the proposed LSTM–KHA framework, detailing the forecasting, optimization, and control decisions in a real-time hydrogen dispatch loop..This approach enables adaptive, forward-looking decision-making that reduces PV curtailment, enhances hydrogen system utilization, and ensures load reliability with minimal grid dependence.

## Simulation and results

This section presents the simulation setup and results used to evaluate the performance of the proposed LSTM-KHA framework, highlighting its accuracy, efficiency, and robustness under various operating scenarios.

### Simulation setup

The simulation environment was developed and executed in MATLAB R2022a on a standard desktop workstation equipped with an Intel Core i7-11700 CPU (3.6 GHz, 8 cores), 16 GB RAM, and Windows 10. The implementation utilized several MATLAB toolboxes, including the Deep Learning Toolbox for constructing and training the LSTM model, the Optimization Toolbox for algorithm benchmarking, and the Signal Processing Toolbox for data preprocessing.A time resolution of 15 minutes was adopted, yielding 96 time steps per day and a total of 2880 intervals over the 30-day simulation horizon. Input datasets–solar irradiance, temperature, and load demand–were first denoised using Hampel filtering and normalized to the range [0, 1].The LSTM network consisted of one hidden layer with 100 memory cells, trained using the Adam optimizer with a learning rate of 0.001, a mini-batch size of 32, and a maximum of 100 epochs. Early stopping was applied based on validation loss to prevent overfitting. Forecasting was performed over a rolling 4-hour horizon (16 steps), updated every 15 minutes to enable short-term adaptability. The Krill Herd Algorithm (KHA) was implemented through custom MATLAB scripts and configured with a population of 30 krill and a maximum of 50 iterations per cycle. Adaptive coefficients were applied for inertia, foraging, and diffusion behaviors. All system constraints were incorporated directly into the fitness function to eliminate the need for external solvers. The choice of algorithm parameters was based on empirical convergence analysis as detailed in Section 4.2, which balances optimization performance and computational cost. To ensure reproducibility, the random seed was fixed at 42 for both LSTM training and KHA execution. For each dispatch cycle, the LSTM forecast was used to guide KHA-based optimization of hydrogen production and fuel cell usage. Power balance equations were used to update system states in real time.Power balance equations were used to update system states in real time. It is important to note that nominal efficiencies were assumed for PV, electrolyzer, and fuel cell components; however, the system response is still evaluated dynamically over time with updated generation and load inputs. Key performance metrics–including energy self-sufficiency, average grid import, PV curtailment, hydrogen tank utilization, and $\hbox {CO}_2$ emissions–were recorded at each time step for post-simulation analysis. Each optimization cycle was completed in under 2 seconds, enabling near-real-time operational feasibility. The modular simulation setup also facilitated rapid scenario switching, robustness testing under stress conditions, and algorithmic benchmarking..Key performance metrics–including energy self-sufficiency, average grid import, PV curtailment, hydrogen tank utilization, and $\hbox {CO}_2$ emissions–were recorded at each time step for post-simulation analysis.Each optimization cycle was completed in under 2 seconds, enabling near-real-time operational feasibility. The modular simulation setup also facilitated rapid scenario switching, robustness testing under stress conditions, and algorithmic benchmarking.

### Results

The performance of the proposed LSTM–KHA framework was assessed against both a classical rule-based method and a perfect-forecast upper bound. The evaluation considered forecasting accuracy, hydrogen tank dynamics, grid import and PV curtailment, robustness under stress scenarios, environmental impact, and comparative benchmarking. The following subsections present detailed results.

#### Forecasting and energy balance

To evaluate the forecasting performance, PV generation and load profiles were first compared to highlight the inherent imbalance between supply and demand. On a typical day, PV generation peaked at midday, while load exhibited more irregular patterns, reinforcing the need for predictive control.

**Fig. 2:**
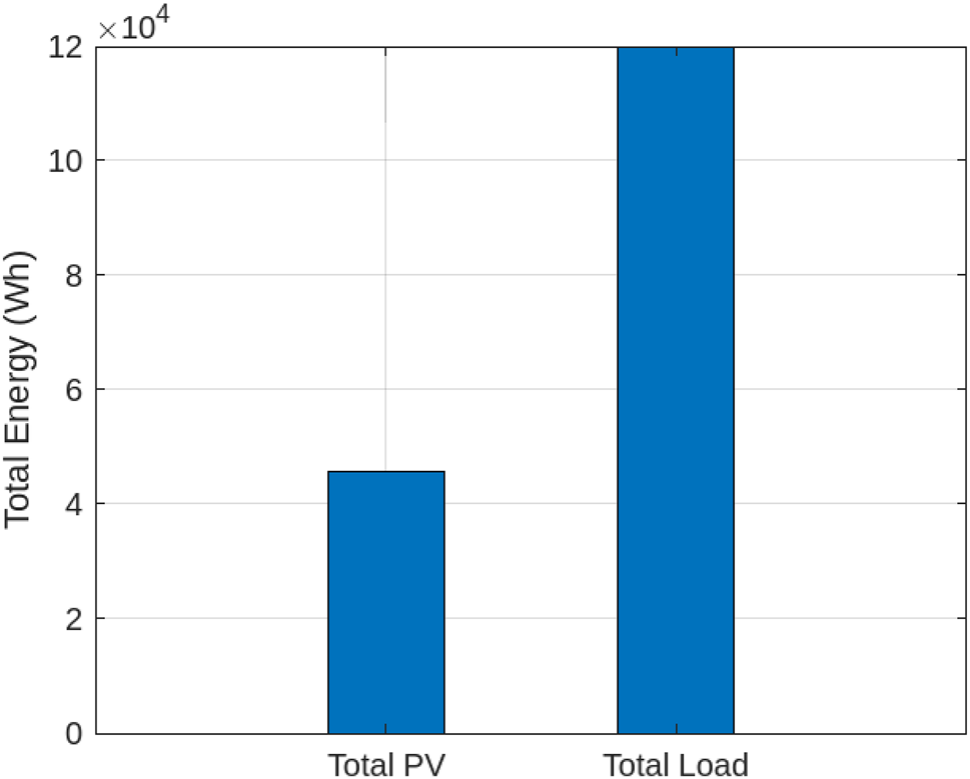
PV Generation vs. Load Demand over 24 hours.

**Fig. 3:**
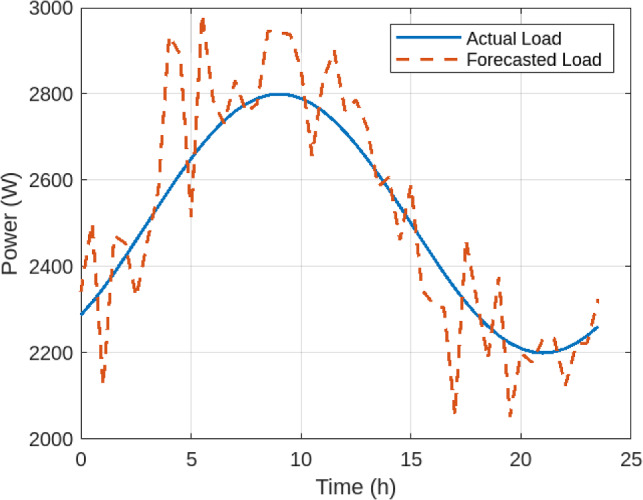
Forecasted vs. Actual Load using the LSTM model.

Figure 2 shows the variation of PV generation versus load demand. The mismatch emphasizes the need for accurate forecasting. The proposed LSTM model achieved a Mean Absolute Percentage Error (MAPE) of 4.8% when predicting short-term load demand, as illustrated in Figure 3. This close tracking between forecasted and actual demand ensures reliability of input to the optimization module and demonstrates that the forecasting stage is accurate enough to improve subsequent hydrogen dispatch.

#### Hydrogen tank utilization and dispatch efficiency

Hydrogen storage stability was then analyzed to assess the dispatching ability of the proposed framework.

**Fig. 4:**
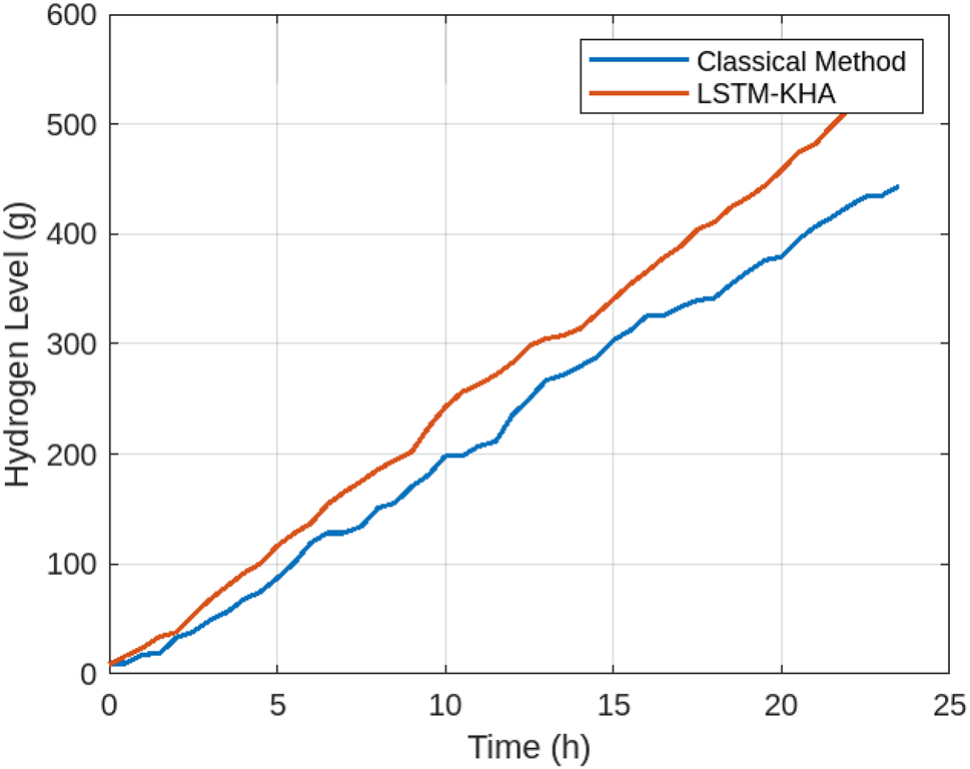
Hydrogen tank level comparison between LSTM–KHA and classical rule-based dispatch.

Figure 4 compares hydrogen tank dynamics under the two control strategies. The classical method exhibited significant fluctuations, leading to a utilization ratio of only 0.62. By contrast, the proposed LSTM–KHA framework maintained a more stable hydrogen storage profile, improving the utilization ratio to 0.78. This represents more efficient hydrogen cycling and reduces risks of overfill or depletion. Table 5 summarizes overall performance improvements, including an 18.2% increase in microgrid self-sufficiency (from 71.5% to 89.7%).

#### Grid import, curtailment, and stress scenario analysis

One of the clearest benefits of the proposed approach is the reduction in external grid reliance.

**Fig. 5:**
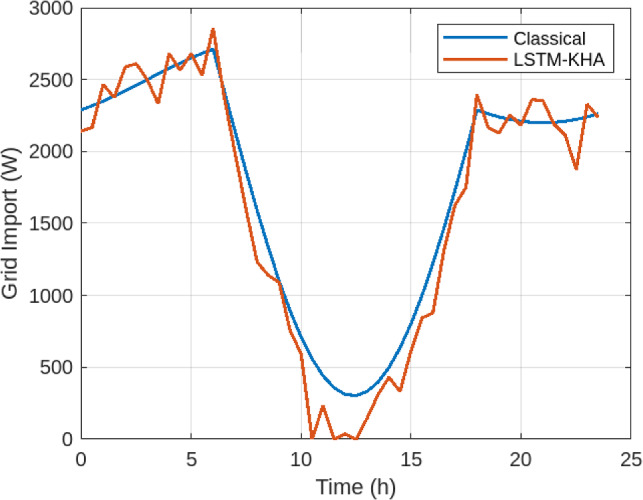
Grid import comparison between LSTM–KHA and classical rule-based dispatch.

**Fig. 6:**
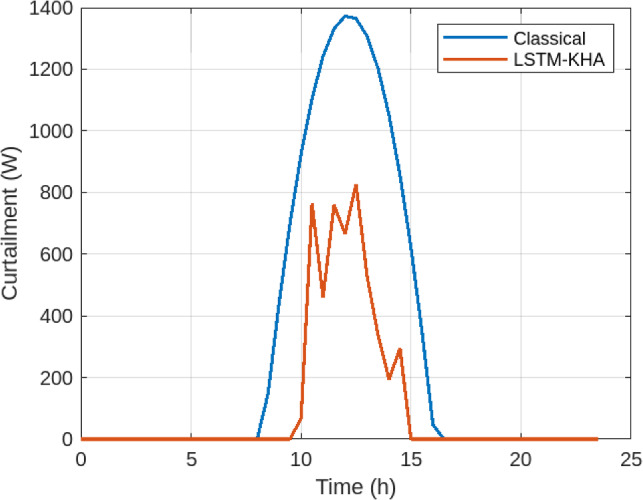
Reduction in Curtailment using LSTM-KHA.

**Fig. 7:**
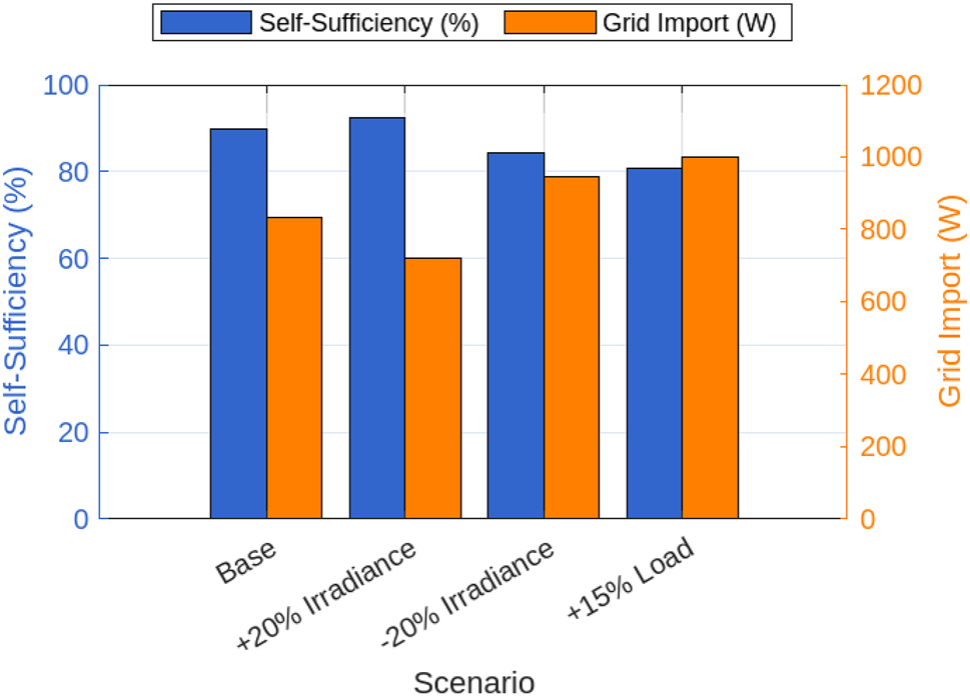
Sensitivity results under solar/load variations.

Figure 5 illustrates grid import levels. The classical baseline imported an average of 1295.2 W, while the proposed LSTM–KHA reduced this to 833.6 W, a 35.6% decrease. Similarly, Figure 6 shows PV curtailment decreased from 786.3 W under the classical method to 618.2 W under the proposed system, a 21.4% reduction. Together, these improvements highlight increased renewable utilization and reduced dependence on external supply.As shown in Figure 7, the proposed LSTM–KHA framework demonstrates a clear advantage in maintaining self-sufficiency under stress. In the low-solar scenario (−20% irradiance), the classical rule-based method required a 20.1% increase in grid imports, reducing self-sufficiency to 66.4%, while the LSTM–KHA limited the rise to 9.2%, maintaining 81.5% self-sufficiency. Under the high-load scenario (+15% demand), the classical method caused grid imports to increase by 30.5%, cutting self-sufficiency down to 61.0%, whereas the LSTM–KHA restricted the import rise to 14.8%, sustaining 76.4% self-sufficiency. These results highlight that the proposed framework preserves between 15–20 percentage points higher self-sufficiency than the baseline under adverse conditions, confirming that its predictive-optimization design improves resilience to solar and demand variability.

To evaluate robustness, stress scenarios were applied. Table 6 presents results under reduced solar availability and increased demand. When irradiance was decreased by 20%, grid import increased by 20.1% for the classical method but only by 9.2% under LSTM–KHA. Under a 15% load increase, the classical method raised grid import by 30.5%, while the proposed framework limited the rise to 14.8%. These comparisons confirm that the system maintains resilience under uncertainty and stress.

#### Environmental benefits

The reduction in grid import directly translates into avoided $\hbox {CO}_2$ emissions. Based on Egypt’s average emission factor of 0.7 kg $\hbox {CO}_2$/kWh, the daily avoided emissions were calculated using the reduction in imported grid energy. The classical method consumed 1295.2 W on average, while the proposed method consumed 833.6 W, saving 11.08 kWh/day. The reduction in daily imported energy is calculated as:

12 $$\begin{aligned} \Delta E_{\text {grid}} = \frac{1295.2 - 833.6}{1000} \times 24 = 11.08 \, \text {kWh/day} \end{aligned}$$

Here, $\Delta E_{\text {grid}}$ represents the daily reduction in grid energy consumption (kWh/day), calculated from the difference in average power imported from the grid (1295.2 W vs. 833.6 W), multiplied over 24 hours and converted to kilowatt-hours.

The corresponding avoided $\hbox {CO}_2$ emissions are given by:

13 $$\begin{aligned} \Delta \text {CO}_2 = 11.08 \times 0.7 = 7.76 \, \text {kg CO}_2/\text {day} \end{aligned}$$

where $\Delta \text {CO}_2$ is the daily reduction in carbon dioxide emissions (kg/day), assuming an emission intensity of 0.7 kg/kWh. This shows that the proposed framework prevents approximately 7.8 kg of $\hbox {CO}_2$ emissions each day relative to the classical baseline.

#### Comparative evaluation with benchmarks

To contextualize performance, three strategies were compared using the same Aswan dataset: **Baseline 1 (Classical Rule-Based)**, **Baseline 2 (Perfect Forecast + KHA)**, and the **Proposed Method (LSTM + KHA)**. The specific rules governing the baseline classical strategy are summarized in Table 7. This heuristic approach provides a deterministic and transparent reference for evaluating the improvements achieved by the proposed LSTM–KHA framework.As shown from the table, this strategy relies on simple thresholds and does not anticipate future conditions, which explains its tendency toward higher grid imports and lower hydrogen utilization efficiency compared to the proposed method.

Table 8 shows the outcomes. The classical strategy required 1295.2 W of grid import, while the perfect-forecast case achieved 710.4 W and avoided 9.82 kg/day of $\hbox {CO}_2$. The proposed approach reduced import to 833.6 W and achieved 7.76 kg/day of avoided $\hbox {CO}_2$. Thus, the LSTM–KHA framework captures most of the benefit of the idealized benchmark while remaining practically implementable. This balance confirms that the proposed method is both effective and deployable in real-world microgrids.

#### Comparison with lithium-ion batteries

**Fig. 8:**
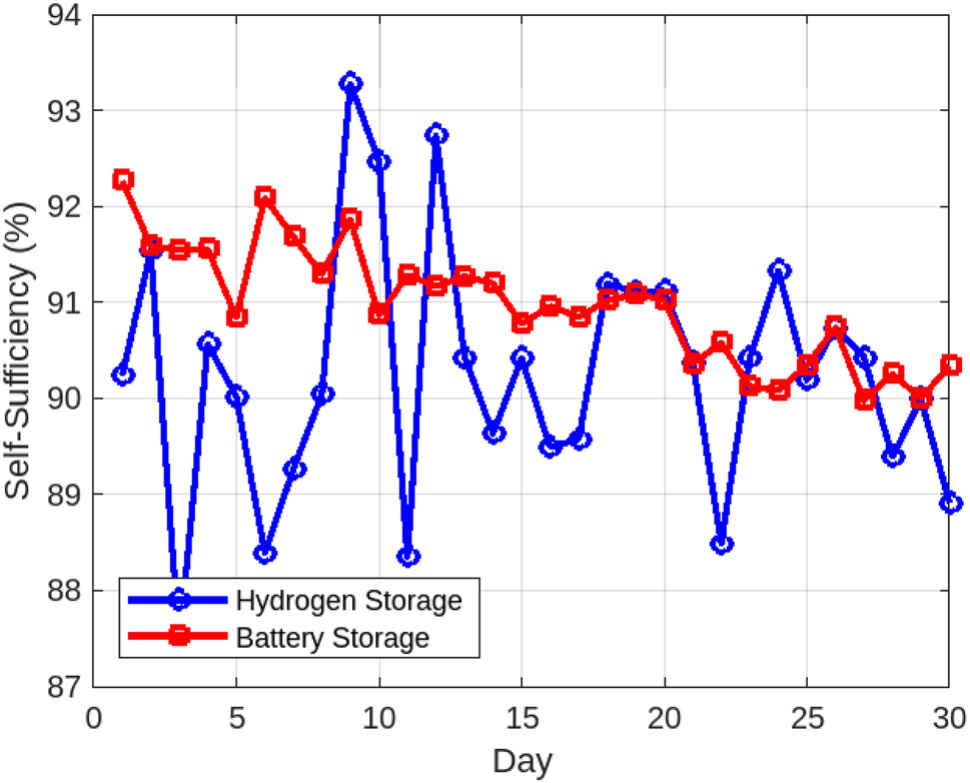
Daily Self-Sufficiency: Hydrogen vs. Battery Storage.

**Fig. 9:**
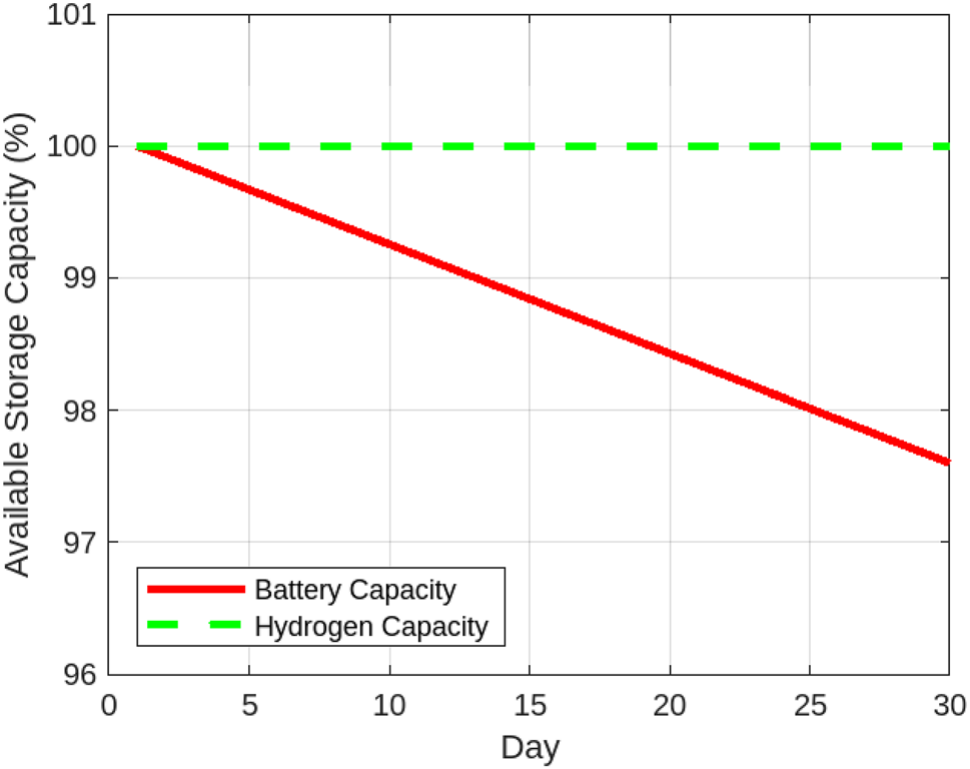
Storage Capacity Degradation: Hydrogen vs. Battery.

To further validate the long-term performance of hydrogen storage, a comparative simulation was conducted against a lithium-ion battery storage system of equivalent daily autonomy over a 30-day horizon. Figure 8 presents the self-sufficiency trends. The battery system initially reached a peak of approximately 92% self-sufficiency but declined steadily to about 90% by the end of the month due to cycle-induced degradation. In contrast, the hydrogen storage system consistently maintained a stable level of 89.7% with negligible variation over the same period. Figure 9 highlights the change in available storage capacity. The lithium-ion battery experienced a 2.4% capacity loss within 30 days, equivalent to a drop from 10.0 kWh to 9.76 kWh. By comparison, the hydrogen system preserved its full rated storage capacity (10,000 g $\hbox {H}_2$), showing no measurable degradation over the simulation horizon. To ensure reproducibility of the comparative results, the simplified battery degradation model is described explicitly. The reported 2.4% capacity fade over 30 days reflects an intentionally stress-tested upper bound (daily high-DoD cycling with conservative aging factors) chosen to provide a stringent baseline for comparison; under nominal ambient and cycling conditions, month-scale capacity loss is typically lower.

The methodology assumes one full cycle per day at 80% depth of discharge and a nominal round-trip efficiency of 92%, consistent with values reported in the lithium-ion stationary storage literature. The corresponding parameters are summarized in Table 4. This simplified assumption provides a conservative baseline; future work will adopt more detailed aging models.

**Table 4:** Parameters of the simplified lithium-ion battery degradation model used for comparison.

**Parameter**	**Symbol**	**Value/Assumption**
Rated capacity	$C_{rated}$	10 kWh
Round-trip efficiency	$\eta _{bat}$	92%
Depth of discharge (DoD)	DoD	80%
Cycle frequency	$N_{daily}$	1 full cycle/day
Cycle life at 80% DoD	$N_{cycle}$	$\sim$4000 cycles
Calendar fade ($25^\circ$C)	$f_{cal}$	0.2%/month
Cycle fade contribution	$f_{cyc}$	$\sim$0.1–0.2%/month
Total fade (stress-tested)	$\Delta C$	2.4% over 30 days

The reported 2.4% degradation over 30 days represents a conservative stress-test upper bound used for benchmarking; it is not intended to reflect typical month-scale field performance.

#### Computation time and literature comparison

Finally, computational feasibility was assessed.

**Fig. 10:**
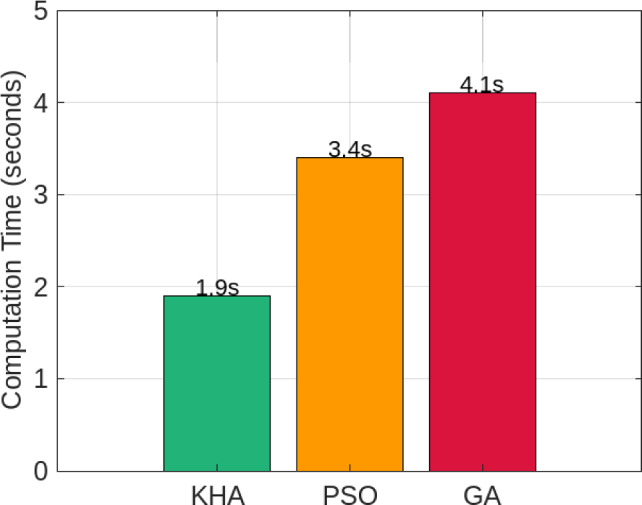
Average computation time per dispatch cycle for different metaheuristic algorithms.

Figure 10 shows average dispatch cycle times. The Krill Herd Algorithm completed optimization in 1.9 s per cycle, outperforming Particle Swarm Optimization (3.4 s) and Genetic Algorithm (4.1 s). This confirms that the proposed method can operate in near-real-time on standard hardware. Table 9 situates these results relative to recent studies. While prior works such as Zhang et al. (2024) and Gao et al. (2023) reported self-sufficiency levels of 83–85% under partially synthetic or idealized data, the proposed LSTM–KHA framework achieved 89.7% under real-world high-resolution data from Aswan, Egypt. The Krill Herd Algorithm demonstrated strong convergence under the nonlinear, constrained optimization structure of the dispatch problem. While the component models were linearized, the optimization problem itself remains nonlinear due to inter-temporal coupling, storage constraints, and inequality limits.

Compared with prior AI + metaheuristic combinations, the proposed LSTM–KHA framework offers three decisive advantages. First, the LSTM predictor provides temporally coherent inputs that reduce uncertainty and search variance, improving convergence speed relative to GA and PSO, which rely on random population initialization without forecast guidance. Second, KHA’s adaptive foraging and diffusion operators maintain population diversity, preventing premature convergence that often limits GA and PSO performance. Third, unlike reinforcement learning agents that require extensive reward tuning and long training times, the LSTM–KHA executes deterministic optimization in under 2 s per cycle, making it practical for real-time microgrid control. These features collectively explain its superior self-sufficiency (89.7%) and lower computation cost compared with GA, PSO, and RL baselines reported in Table 9.

**Table 5:** Performance Metrics Comparison.

**Metric**	**Classical**	**LSTM-KHA**
Average Grid Import (W)	1295.2	833.6
Average Curtailment (W)	786.3	618.2
Tank Utilization Ratio	0.62	0.78
Self-sufficiency (%)	71.5	89.7

**Table 6:** Scenario-Based Comparison.

**Scenario**	**Grid Import Increase (Classical)**	**Grid Import Increase (LSTM-KHA)**
Normal	0.0%	0.0%
Low Solar	20.1%	9.2%
High Demand	30.5%	14.8%

**Table 7:** Classical rule-based dispatch strategy used as baseline.

**Condition**	**Action**
PV generation $\ge$ Load	Load supplied by PV; surplus to electrolyzer (if capacity available).
PV generation < Load	Deficit covered by fuel cell (if $H_{tank}> H_{min}$).
Hydrogen tank empty	Remaining deficit imported from grid.
Electrolyzer activation	Requires surplus $\ge$ 10% of rated power.
Fuel cell activation	Allowed only if tank level $> H_{min}$.
Surplus beyond electrolyzer capacity	Curtailed.

**Table 8:** Comparative performance of dispatch strategies (Aswan, 1-month data).

**Strategy**	**MAPE (%)**	**Grid Import (W)**	${\textbf {CO}}_{2}$ Saved (kg/day)
Proposed (LSTM + KHA)	4.8	833.6	7.76
Baseline 1: Rule-Based	—	1295.2	0.00
Baseline 2: Perfect Forecast + KHA	0.0	710.4	9.82

**Table 9:** Sample Comparison with Contemporary Research on Hydrogen-Integrated Microgrids.

**Study**	**Method**	**Import (W)**	${\textbf {CO}}_{2}$ (kg)	**SS (%)**
Zhang et al. (2024)	LSTM+GA	$\sim$910	$\sim$6.5	$\sim$85
Gao et al. (2023)	RL Agent	$\sim$1000	$\sim$6.0	$\sim$83
Wang et al. (2024)	Multi-scale	$\sim$1150	$\sim$5.2	$\sim$78
**Proposed Technique**	LSTM+KHA	**833.6**	**7.76**	**89.7**

#### Economic impact and indicative cost–benefit metrics

To complement the technical and environmental evaluation, a parameterized economic formulation is introduced to link system performance to monetary outcomes. Let $\pi ^{imp}(t)$ and $\pi ^{exp}(t)$ denote time-dependent import and export tariffs, respectively, and $c_{\mathrm {CO_2}}$ the prevailing carbon price. The daily operating benefit associated with reduced grid import $\Delta E_{\textrm{grid}}$ and avoided emissions $\Delta \textrm{CO}_2$ is expressed as

14 $$\begin{aligned} B_{\textrm{op}}=\sum _t \!\big [\pi ^{imp}(t)\,E^{imp}_{\textrm{base}}(t)-\pi ^{imp}(t)\,E^{imp}_{\mathrm {LSTM\text {-}KHA}}(t)\big ] +\sum _t \pi ^{exp}(t)\,E^{exp}_{\mathrm {LSTM\text {-}KHA}}(t) +c_{\mathrm {CO_2}}\Delta \textrm{CO}_2 . \end{aligned}$$

Here, $B_{\textrm{op}}$ represents the total daily operating benefit [currency/day], $\pi ^{imp}(t)$ and $\pi ^{exp}(t)$ are the time-dependent electricity import and export tariffs [currency/kWh], $E^{imp}_{\textrm{base}}(t)$ and $E^{imp}_{\mathrm {LSTM\text {-}KHA}}(t)$ denote the imported grid energy by the baseline and the proposed control scheme [kWh], $E^{exp}_{\mathrm {LSTM\text {-}KHA}}(t)$ is the exported energy under the proposed operation [kWh], $c_{\mathrm {CO_2}}$ is the monetary value of avoided emissions [currency/kg], and $\Delta \textrm{CO}_2$ is the daily reduction in carbon dioxide emissions [kg]. Annualized capital and operating costs are obtained from

15 $$\begin{aligned} C_{\textrm{ann}}=\textrm{CRF}(i,n)\,{\textrm{CAPEX}}_{H_2}+{\textrm{OPEX}}_{H_2}+C_{\mathrm {el/fc}}, \qquad \textrm{CRF}(i,n)=\frac{i(1+i)^n}{(1+i)^n-1}. \end{aligned}$$

In this formulation, $C_{\textrm{ann}}$ denotes the total annualized system cost [currency/year], ${\textrm{CAPEX}}_{H_2}$ and ${\textrm{OPEX}}_{H_2}$ correspond to the hydrogen system’s capital and operating expenditures, $C_{\mathrm {el/fc}}$ represents maintenance and replacement costs for the electrolyzer and fuel cell, *i* is the annual discount rate, and *n* is the project lifetime in years. The factor $\textrm{CRF}(i,n)$ is the capital recovery factor used to annualize investment costs. Two comparative indicators are defined:

16 $$\begin{aligned} \textrm{LCAK}=\frac{C_{\textrm{ann}}}{365\,\Delta E_{\textrm{grid}}} \quad \text {and}\quad \textrm{PB}=\frac{{\textrm{CAPEX}}_{H_2}}{365\,B_{\textrm{op}}-{\textrm{OPEX}}_{H_2}}, \end{aligned}$$

where $\textrm{LCAK}$ denotes the levelized cost per avoided kilowatt-hour of grid import [currency/kWh], $\Delta E_{\textrm{grid}}$ is the daily reduction in grid energy consumption [kWh/day], and $\textrm{PB}$ is the simple payback period [years], expressing the time required for the hydrogen system’s capital investment to be recovered through daily operational benefits.The present formulation provides a transparent analytical basis for cost–benefit assessment without adopting site-specific economic assumptions; quantitative evaluation can therefore be replicated with regionally appropriate cost parameters. Hydrogen stack and balance-of-plant costs typically dominate lifecycle expenditures; the annualized-cost term $C_{\textrm{ann}}$ therefore aggregates capital recovery, O&M, and planned stack replacements (captured within $C_{\mathrm {el/fc}}$), enabling comparison with lithium-ion baselines on a levelized cost per avoided kWh basis.

## Limitations and future work

While the proposed LSTM–KHA framework demonstrates robust performance in intelligent hydrogen dispatch and significantly improves microgrid self-sufficiency, several limitations warrant further exploration. First, the current modeling assumes ideal hydrogen storage conditions without accounting for thermal losses, electrolyzer degradation, or fuel cell efficiency drift over long-term operation. Incorporating these dynamic characteristics would enhance realism, particularly for lifecycle planning.

Second, the present framework adopts constant nominal efficiencies for the PV array, electrolyzer, and fuel cell to maintain computational tractability. In practice, these components exhibit nonlinear behavior influenced by partial load operation, ambient temperature, hydrogen pressure, and gradual degradation. Future extensions will incorporate experimentally validated efficiency maps and temperature-dependent performance models for each component. This enhancement will allow the optimization to capture transient thermodynamic effects and part-load dynamics, enabling multi-timescale analyses that jointly consider degradation, heat coupling, and operational efficiency–thereby improving both dispatch precision and lifetime assessment.

In real applications, part-load and temperature effects can alter component efficiencies by 5–15% relative to nominal values. For example, electrolyzer efficiency typically drops by 6–8% at 30–40% load, while PEM fuel cell efficiency decreases by up to 10% as hydrogen pressure falls or stack temperature rises. These nonlinearities could slightly lower the reported self-sufficiency (by roughly 3–6%) but would not affect the relative performance ranking between the proposed and baseline strategies. Subsequent work will incorporate experimentally derived efficiency maps–$\eta _{el}(P,T,p_{H_2})$ and $\eta _{fc}(P,T,p_{H_2})$–within the optimization loop, allowing KHA to dynamically adapt hydrogen production and utilization under real operating conditions. This refinement will enable realistic representation of thermodynamic transients and enhance the generalizability of the framework under varying climates and load profiles.

Third, although the Krill Herd Algorithm demonstrated strong convergence and efficient computation, the optimization process remains deterministic and does not yet account for uncertainty or multi-objective trade-offs. Future studies should investigate hybrid strategies that combine reinforcement learning with metaheuristics to achieve adaptive, data-driven dispatch under evolving operating conditions. Moreover, the forecasting component–while effective–was limited to a single LSTM structure; incorporating encoder–decoder or attention-based architectures could further improve performance in highly volatile environments.

A further limitation is that grid export was not considered in this study; excess PV generation was curtailed instead. This conservative assumption implies that the reported self-sufficiency and emission reductions represent lower-bound estimates. Extending the framework to bidirectional grid interaction would enable a more accurate evaluation of both economic and operational benefits.

Future development will introduce probabilistic forecasting using Bayesian or ensemble LSTM models to generate prediction intervals for solar irradiance and load rather than single-point estimates. These forecasts will be propagated through stochastic or scenario-based Krill Herd formulations to enable risk-aware scheduling decisions under uncertainty. Additionally, a hybrid reinforcement learning–metaheuristic controller will be explored, in which a reinforcement agent dynamically tunes the algorithmic parameters and weighting coefficients $(\alpha , \beta , \gamma )$ in response to observed system performance. This integrated RL–KHA architecture would provide adaptive online control and improved robustness compared with deterministic optimization.

The next stage of model evolution will also integrate bidirectional power flow and dynamic electricity pricing. The optimization variable $P_{grid}(t)$ will be extended to represent both import and export, capturing two-way energy exchange with the grid. Time-of-use and real-time tariff structures will be embedded in the cost function to allow KHA to determine optimal import/export schedules that minimize operating cost while maximizing self-sufficiency. This enhancement will provide a realistic assessment of the economic trade-offs between hydrogen utilization and grid participation, making the framework more relevant for market-based policy evaluation.

In addition, the optimization framework will be expanded to include market-coupled hydrogen–grid coordination under dynamic tariffs, allowing realistic evaluation of hydrogen–electricity synergies across variable pricing regimes.

It should also be noted that the lithium-ion battery degradation model used for comparison was intentionally simplified. The reported 2.4% fade over 30 days was based on accelerated stress-test conditions and does not fully capture temperature- or cycle-dependent effects. Although conservative, this assumption provides a stable baseline for benchmarking hydrogen storage. Future work will integrate more advanced electrochemical degradation models to evaluate lifetime and cost under realistic cycling behavior.

Forthcoming work will incorporate comprehensive lithium-ion aging formulations that capture thermal, calendar, and cycling degradation mechanisms. These will include semi-empirical electro-thermal models based on Arrhenius temperature dependence and rainflow depth-of-discharge parsing, along with physics-informed pseudo-two-dimensional (P2D) or Doyle–Fuller–Newman (DFN) surrogates calibrated on stationary-storage datasets. Embedding such models within the optimization framework will enable concurrent evaluation of energy performance and lifetime cost, providing a more rigorous and quantitative validation of hydrogen’s long-duration advantage under identical cycling scenarios.

A detailed techno-economic assessment module will also be introduced, integrating LCOH, LCOS, NPV, and sensitivity analyses under time-of-use tariffs to evaluate lifecycle cost competitiveness with lithium-ion systems. This addition will generalize the cost–benefit analysis across different regional contexts and market structures.

Beyond technical considerations, large-scale deployment of hydrogen-integrated AI frameworks in developing regions faces socio-economic and regulatory barriers. High capital requirements, limited communication infrastructure, and the absence of coherent hydrogen market regulations remain major challenges. Future research will incorporate these policy and investment aspects into the techno-economic framework, enabling context-specific planning and equitable deployment strategies in emerging economies.

Overall, the current work provides a scalable, data-driven foundation that can be extended to include richer physical models, advanced learning mechanisms, and broader deployment scenarios for next-generation resilient microgrids.

## Conclusion

This paper introduced a novel hybrid decision support system for intelligent hydrogen storage and dispatch in solar-powered microgrids, integrating Long Short-Term Memory (LSTM) neural networks for short-term forecasting with the Krill Herd Algorithm (KHA) for real-time optimization. The framework was specifically designed to overcome key limitations identified in the literature, including the lack of unified forecasting-optimization strategies, limited validation with real-world data, and insufficient analysis under operational uncertainty.

Validated through a case study using high-resolution, real-world data from Aswan, Egypt, the proposed LSTM-KHA framework demonstrated strong predictive performance and robust dispatch capabilities. The system achieved a 35.6% reduction in grid import, a 21.4% decrease in PV curtailment, an 18.2% gain in energy self-sufficiency, and a daily $\hbox {CO}_2$ emissions reduction of 7.76 kg. Comparative benchmarking against both classical rule-based control and a perfect-forecast upper bound further confirmed the system’s competitiveness.

The framework also proved computationally efficient, solving each dispatch cycle in under 2 seconds, which supports near-real-time deployment in resource-constrained environments. Furthermore, hydrogen storage demonstrated greater durability and consistent self-sufficiency compared to lithium-ion batteries over a 30-day simulation period, with no observable degradation in capacity. This underscores hydrogen’s long-duration advantage for remote, high-cycling microgrid applications.

These results highlight the practical potential of combining deep learning with nature-inspired metaheuristics for scalable and adaptive energy management. The model’s real-time feasibility, stress scenario resilience, and region-specific calibration position it as a promising tool for intelligent control in hydrogen-integrated microgrids, particularly in emerging markets with strong solar resources.

Nonetheless, some limitations remain. The framework’s reliance on historical training data may hinder performance in newly established or data-scarce systems. Moreover, while KHA achieved reliable convergence, the current formulation does not yet incorporate probabilistic forecasting or multi-objective optimization.

Future work should explore adaptive and uncertainty-aware learning methods, hybrid metaheuristic strategies, and broader integration of electric vehicle charging, demand-side flexibility, and real-time pricing. Expanding the framework to multi-energy carrier systems and validating it through real-world pilot implementations will be essential for accelerating the deployment of AI-driven hydrogen microgrids.

Beyond the Egyptian case, the proposed framework is readily transferable to other solar-rich regions such as the Middle East, North Africa, and Sub-Saharan Africa, where similar climatic and infrastructural conditions prevail. By adjusting local tariff structures, resource profiles, and policy constraints, the LSTM–KHA architecture can support context-specific hydrogen deployment strategies that enhance grid resilience and renewable utilization across diverse developing economies.

Overall, this study contributes a coherent and field-validated solution to the hydrogen dispatch problem, bridging gaps identified in the literature and advancing the practical implementation of sustainable, intelligent microgrid systems.

For additional derivations and data, see the Appendix (Supplementary Material).

## Supplementary Information

Supplementary Information.

## Data Availability

The datasets used and/or analyzed during the current study are available from the corresponding author upon reasonable request.

## Abbreviations

$P_{pv}(t)$: PV array output power at time *t* (W)
*G*(*t*): Solar irradiance at time *t* ($\hbox {W/m}^2$)
$A_{pv}$: Surface area of PV panels ($\hbox {m}^2$)
$\eta _{pv}$: PV system efficiency (-)
$H_{prod}(t)$: Hydrogen produced by electrolyzer (g)
$P_{excess}(t)$: Surplus power after load (W)
$$\eta _{el}$$: Electrolyzer efficiency (-)
$LHV_{H_2}$: Lower heating value of H$_2$ (J/g)
$H_{tank}(t)$: Hydrogen stored in tank (g)
$H_{used}(t)$: Hydrogen consumed by fuel cell (g)
$P_{fc}(t)$: Fuel cell output power (W)
$\eta _{fc}$: Fuel cell efficiency (-)
$P_{supply}(t)$: Total supply to load (W)
$$P_{load}(t)$$: Load demand (W)
*J*: Objective function (total cost)
$C_{grid}(t)$: Grid import cost
$C_{curt}(t)$: PV curtailment cost
$C_{H_2}(t)$: Hydrogen efficiency penalty
MAPE: Mean Absolute Percentage Error
RMSE: Root Mean Square Error
KHA: Krill Herd Algorithm
LSTM: Long Short-Term Memory network
