# Supplementary material for: Artificial intelligence powered intelligent energy management framework for hydrogen storage and dispatch in smart microgrids
Source: Sci Rep. 2025 Nov 18;15:40394. doi: 10.1038/s41598-025-24408-7 (PMC12627455; doi:10.1038/s41598-025-24408-7)
Supplement: Supplementary file 1 — Supplementary Information. [file 41598_2025_24408_MOESM1_ESM.pdf]

Appendix: Representative Samples from the Aswan Dataset

The following tables present representative samples of the processed solar and load data used in the simulation study. Table 1 corresponds to a typical summer day, while Table 2 illustrates a winter day, both with 15-minute intervals representative of actual operating conditions in Aswan, Egypt.

Table 1: Representative Sample – June 21, 2022 (Summer Day)

**Timestamp**	**GHI (W/m^2)**	**Temperature (°C)**	**Load (W)**
06:00	0	28.1	418
08:00	529.9	32.6	475
10:00	857.2	36.5	510
12:00	1000	40.0	532
14:00	857.2	39.5	520
16:00	529.9	36.0	610
18:00	153.2	32.1	670
20:00	0	29.4	705

Table 2: Representative Sample – December 21, 2022 (Winter Day)

**Timestamp**	**GHI (W/m^2)**	**Temperature (°C)**	**Load (W)**
06:00	0	16.5	410
08:00	235.4	20.2	455
10:00	520.1	24.0	480
12:00	660.3	26.5	498
14:00	520.1	25.2	490
16:00	235.4	21.0	600
18:00	50.0	18.1	650
20:00	0	17.2	690
